# Considering the Social and Economic Sustainability of AI

**DOI:** 10.1007/s11948-025-00544-1

**Published:** 2025-07-24

**Authors:** Rosalie Waelen, Aimee van Wynsberghe

**Affiliations:** Sustainable AI Lab, Institute for Science and Ethics, University of Bonn, Bonner Talweg 57, 53113 Bonn, Germany

**Keywords:** Artificial intelligence, AI ethics, Sustainability, Sustainable AI, Decolonial AI, Ideology, Critical AI studies

## Abstract

In recent years, the notion of ‘sustainable AI’ has emerged as a new topic within the wider debate on artificial intelligence (AI). Although sustainability is usually understood as having three dimensions – the environment, society, and the economy – the debate on sustainable AI, so far, is characterized by a narrow focus on the environmental sustainability of AI. In this article, it is argued that the debate on sustainable AI should not only be about AI’s environmental costs, but also incorporate social and economic concerns. More precisely, the article shows that AI’s environmental impact is often connected to important social and economic issues. Through an analysis of existing literature on the sustainability of AI, it is demonstrated that ethical and philosophical arguments about the importance of considering the environmental costs of AI apply just as well to the social and economic dimensions of AI development. The aim of this article is thus to present a broader understanding of sustainable AI; one that includes all three pillars of sustainability and acknowledges the interdependence between AI’s environmental, social and economic costs.

## Introduction

Pioneered by the work of Strubell et al. (2019), *The AI Now Institute* (Crawford & Joler, 2018; Dobbe & Whittaker, 2019), Bender and colleagues (2021), Crawford (2021), Brevini (2022), and others, attention for the environmental costs and material reality of AI is growing. While AI has long been seen as something untouchable that exists only ‘in the cloud’, it is now increasingly acknowledged that AI has a material dimension (Brevini, 2022; Strubell et al., 2019). This material dimension first of all has to do with the minerals and other materials sourced for the hardware needed to store data and run models. Secondly, the datacenters involved in storing data and running models consume significant amounts of energy and water.

Research on the sustainability of AI, so far, has been predominantly concerned with this material cost of AI, that is, the environmental sustainability of AI. The argument presented in this article is that focusing solely on the environmental sustainability of AI, when talking about ‘sustainable AI’, is too narrow. The concept of sustainability is commonly understood as having three dimensions: environmental, social, and economic sustainability (Purvis et al., 2019). We argue that these three pillars also apply in the AI context, because there are not only environmental costs and considerations involved throughout AI’s lifecycle, but also social and economic ones. Moreover, the environmental, social, and economic dimensions of the AI lifecycle are thoroughly interdependent. Therefore, we argue, the notion ‘sustainable AI’ should always refer to all three pillars of sustainability.

Our argument for including the social and economic dimensions of AI in the notion of ‘sustainable AI’, is developed through a discussion of recent ethical and philosophical literature on sustainable AI. The structure of the article is as follows. Section “The Two Branches of Sustainable AI” discusses the distinction between AI *for* sustainability and the sustainability *of* AI, which was introduced by (Van Wynsberghe, 2021) and further developed by (Falk & Van Wynsberghe, 2023). We show that both of these branches of sustainable AI apply just as well to social and economic costs involved in the development of AI. Section “Repairing AI” analyzes the notion of ‘repairing AI’, put forward by (Stone & Van Wynsberghe, 2024), in relation to the hidden labor involved in AI as well as the western, White, male, and techno-solutionist views and values reflected in AI. Section “AI Ideology” focuses on the notion of AI ideology (Lindgren, 2023; Schütze, 2024) and demonstrates the interrelation between the three pillars of sustainability, by arguing that the different costs involved in AI development are the direct consequence of prioritizing economic gains. Section “A Structural Turn” discusses how the concept of ‘sustainable AI’ relates to the so-called ‘structural turn in AI ethics’ (Bolte & Van Wynsberghe, 2024) and how ‘sustainable AI’ relates to other notions in the AI ethics debate, such as ‘trustworthy AI’, ‘responsible AI’, ‘human-centered AI’ and ‘explainable AI’. Section “Conclusion” concludes the article by stressing once more the need for a broader understanding of the notion ‘sustainable AI’ in future research, public debate, and policy making. It is only through a broader understanding of sustainable AI, as we argue for here, that the global AI infrastructure can come close to a sustainable one.

## The Two Branches of Sustainable AI

AI research is mostly concerned with sustainability as a goal which AI can serve. Indeed, AI methods and applications can be used to accelerate and/or achieve the UN’s Sustainable Development Goals (SDGs) or other types of sustainability-related goals (Nishant et al., 2020). To give an example: in the field of agriculture, computer vision systems can be used to monitor crops, which helps to optimize the harvesting process and reduce waste (Kakani et al., 2020). While using AI to make societal infrastructures and processes more environmentally friendly is a noble goal, Van Wynsberghe (2021) points out that it is important that developers, ethicists, and policy makers also start paying attention to the substantial environmental cost of AI systems. AI may be able to support environmental sustainability, but at the same time, developing and using AI also has a significant ecological footprint, among other reasons because of the massive datacenters that are needed to train and run AI models. Given this sharp contrast between using AI for sustainability-related goals, on the one hand, and considering the environmental costs of developing and using AI, on the other hand, Van Wynsberghe (2021) proposes to use a clear distinction between ‘AI *for* sustainability’ and the ‘sustainability *of* AI’, when speaking of ‘sustainable AI’.

Van Wynsberghe (2021) defines sustainable AI, which they argue should be the new and primary focus of AI ethics, as follows:

Sustainable AI is a movement to foster change in the entire lifecycle of AI products (i.e. idea generation, training, retuning, implementation, governance) towards greater ecological integrity and social justice. As such, Sustainable AI is focused on more than AI applications; rather, it addresses the whole sociotechnical system of AI. (p. 217)

Already included in this definition is a concern for not only the ecological dimension of the AI lifecycle, but also its social dimension. However, in their discussion of the sustainability of AI, it is only the environmental cost of AI development and use that Van Wynsberghe (2021) pays attention to. In other words, the author’s case for putting the sustainability of AI at the core of the AI ethics debate does not speak against including considerations for the social and economic sustainability of AI. However, we think that these considerations are thus far not sufficiently developed in their, or the field’s, work on the sustainability of AI.

The focus on the environmental cost of AI is not only present in Van Wynsberghe’s^ 2021 ^piece that introduces the distinction between AI *for* sustainability and the sustainability *of* AI, their later work on sustainable AI is also characterized by this focus on environmental sustainability. For instance, Robbins & Van Wynsberghe (2022) describe the field of sustainable AI as aimed at “addressing the environmental justice issues associated with AI throughout its lifecycle” (p. 31) and “about understanding and measuring the environmental impact of developing and using AI” (p. 32). Another example is a 2023 research paper by Falk and Van Wynsberghe, which is aimed at further developing the notion of sustainable AI, building on the distinction between AI *for* sustainability and the sustainability *of* AI. In this paper, it is argued that “AI for Sustainability should include both an action that contributes to a sustainable end goal as well as an investigation of the sustainability issues of the AI system itself” (Falk & Van Wynsberghe, 2023, p. 22). After all, if the environmental cost of AI turns out to be higher than the gain of AI for sustainability initiatives, those initiatives are not quite as beneficial as they are thought or made out to be. However, although the aim of the authors is to further flesh out the notion of sustainable AI, it does so only with respect to the environmental cost of AI and the use of AI for environmental sustainability.

The UN’s Sustainable Development Goals and the notion of sustainability are not limited to environmental aspects and concerns. Sustainability is most commonly understood as having three pillars: an environmental, societal, and economic one (Purvis et al., 2019). In line with this is the fact that there are not only AI initiatives aimed at improving environmental sustainability, but also ‘AI for good’ or ‘AI for social good’ initiatives that are either directly or indirectly linked to the UN’s social and economic Sustainable Development Goals – such as no poverty (SDG 1), quality education (SDG 4), and gender equality (SDG 5) (see for example Vinuesa et al., 2020). So, we insist, the label ‘AI *for* sustainability’ must be understood as referring not only to the use of AI for environmental ends, but also to the use of AI for social and economic good.

Similarly, when we speak of the ‘sustainability *of* AI’, it can imply an investigation into the environmental costs of AI across its entire lifecycle, but also an assessment of the social costs and economic considerations involved in the development and use of AI. The lens of sustainability requires us to consider the entire lifecycle of AI, not only its use phase. Types of labor included in the lifecycle of AI range from the mining of minerals for the production of chips, developing the hardware that supports datacenters, running datacenters, gathering or scraping data, labeling and filtering data, developing and implementing models, to the processing of electronic waste.^1^ Throughout this lifecycle, there are not only a variety of material costs involved, but also various social and economic costs. These socio-economic costs have to do with the fact that, along the entire lifecycle of AI, human labor is involved and local communities and economies are affected.

Let us briefly outline the main socio-economic costs related to the development and infrastructure of AI, i.e., the AI lifecycle. A first example has to do with the *health risks* involved for both AI workers and local communities along the AI development cycle. For instance, there are various health hazards associated with the mining of minerals. As already pointed out, a wide variety of minerals – such as lithium, aluminium, copper, and gold – are needed to create GPU’s, chips, and batteries, which in turn support data servers and centers (Crawford & Joler, 2018). Miners are confronted with many different risks while mining these minerals – such as work accidents, stress, and exposure to dust and toxins (Stewart, 2020). The health of communities living near mines is also at risk, because the mining can contaminate people’s drinking water (Adeel et al., 2023; Mensah & Tuokuu, 2023) or deplete water resources. The creation of chips, the next step in the AI lifecycle, also causes water depletion (Belton, 18 September 2021). This not only deprives local communities of access to drinking water, but also puts their living environment and livelihoods at risk, because of the impact of polluted drinking water and water depletion on agriculture. These issues show clearly that the environmental, social, and economic costs of AI are deeply intertwined.

Another example of social costs involved in the development of AI has to do with the *economic and legal risks* that AI workers face. On top of the health risks involved, many hidden forms of labor in AI are also precarious because of low wages, a lack of job security, and the absence of legal protection for workers (Fuchs, 2014; Muldoon et al., 2024). One type of labor involved in AI, that is slowly receiving recognition, is the labeling and filtering of data by so-called ‘data workers’ or ‘gig workers’ (see for example Chen, 28 March 2019; Gray & Suri, 2019; Miceli & Posada, 2022; Muldoon et al., 2024; Perrigo, 18 January 2023). These workers are either crowdsourced over online platforms (part of the platform economy), or working for Business Process Outsourcing companies (BPOs). These digital platforms and BPOs function as an intermediate or facilitator between workers and customers. While workers at BPOs usually still have short-term contracts, platform workers are not employed at all; they are independent workers who get paid per gig. The field of AI relies heavily on these types of workers, to take care of tasks that are not (yet) automatable. An example of such tasks is the labeling of the images that are used to train computer vision models, or giving feedback to chatbots to improve their output.

Gray and Suri (2019) have described data workers as ‘ghost workers’, because their labor and involvement in the AI industry is largely hidden and unknown. Altenried (2020) further explains that platform labor can be deemed ‘hidden’ for multiple reasons:

The labour of crowdworkers is hidden in various ways. Mostly, it takes place outside of public spaces, and often in private homes. It is geographically distributed and less visible than most other forms of labour. It also takes place outside of the reach of many forms of labour regulation and legislation and traditional forms of labour conflict. Furthermore, it is hidden behind the magic of algorithms: Many of the work done on digital platforms is masked as software. Much of the work that is done by crowdworkers is thought to be already automated. (Altenried, 2020, p. 157)

BPOs and digital platforms of course form an opportunity for economies that struggle with high unemployment, as well as for individuals that find themselves excluded on the traditional labor market (e.g. women with care duties, or migrants that have not yet mastered the language of their new countries – examples discussed by Altenried, 2020). However, the freedom and flexibility that this data work promises, comes at the price of precarity, vulnerability, and a loss of autonomy (Anwar & Graham, 2021; Christiaens, 2022; Graham et al., 2017).

Finally, from the examples discussed in this section, it should become obvious that the environmental and social costs of AI are often accompanied by economic costs and considerations as well. First of all, as mentioned above, the extent to which the AI industry provides economic opportunities for workers in the Global South, and vulnerable societal groups everywhere, needs to be considered. However, the fact that the tech industry provides an opportunity for these workers does not excuse exploitative labor practices. Secondly, the environmental and social costs of AI can be seen as the consequence of economic opportunism on the side of the tech industry (we will discuss this issue in more detail in Sect. “AI Ideology”). Thirdly, the AI industry challenges certain economies in the Global South either by taking away workers from other industries or by damaging industries (e.g. agriculture) as a result of AI’s environmental impact (Belton, 18 September 2021). What this shows is that the three pillars of sustainability should not only each play a role when thinking about the sustainability of AI, as we argue in this article, the three pillars are also strongly *interdependent*.

## Repairing AI

The social and economic costs of AI development, listed in Sect. “The Two Branches of Sustainable AI”, require us to revise yet another philosophical argument made about sustainable AI. In a recent article, Stone and Van Wynsberghe (2024) argued that AI is in need of *repair*. By linking the notion of ‘sustainability’ to the concept of ‘repair’, the authors try to further refine the notion of sustainable AI conceptually. They argue that AI *for* sustainability can be understood as using AI for acts of repair (e.g. reducing emissions), and that the sustainability *of* AI is all about repairing AI infrastructures itself. The authors argue that, as an increasingly important infrastructure, AI first of all needs repair in the sense that its environmental costs need to be addressed. In other words, AI’s infrastructures need to be transformed for the sake of environmental justice. Secondly, the argue that we need to repair how we think about AI. This sense of repairing AI should be understood in a metaphorical or figurative sense. The common image of AI, as being something untouchable that exists only ‘in the cloud’, should be replaced by one that reflects its true materiality.

Although Stone and Van Wynsberghe make a compelling case for repairing AI, both literally and figuratively speaking, their view on what it is about AI that is in need of repair remains limited to AI’s materiality and the environmental costs of AI. However, these are not the only systemic and hidden problems in AI that are in need of repair. In analogy with Stone and Van Wynsberghe’s discussion about repairing AI’s material infrastructure for the sake of environmental justice and sustainability, we argue here that it is also the social and economic infrastructures associated with AI that need to be repaired, for the sake of social justice and global justice. Stone and Van Wynsberghe (2024) point out that AI has a hidden material reality and, so the authors argue, repairing AI involves acknowledging this hidden materiality. In analogy, we argue that AI has hidden social and economic realities that need to be acknowledged and demand repair.

A first, social issue that calls for repair is the aforementioned hiddenness and precarity of the human labor involved throughout AI’s lifecycle. AI is often seen as something automatic and autonomous, that will make human labor superfluous. This image of AI and automation tactfully conceals the amount and variety of human labor that is involved in the development and use of AI products. As we pointed out in the previous section, human labor is needed throughout the entire AI lifecycle. For instance, there is human labor involved in mining the minerals needed to create chips in AI hardware, in the production of those chips, in the running and maintenance of datacenters, in the data collection and data annotation, in developing and finetuning AI models, and in processing electronic waste. Not to mention the commercial aspects of the AI industry – jobs that are also performed by human workers. Those directly involved in developing and implementing AI models – like data scientists, machine learning engineers, and AI architects – are known to receive generous compensations for their efforts, while workers elsewhere along the AI lifecycle are likely to be confronted with precarious working conditions. The hiddenness and precarity of these types of AI labor go against ideals of social and distributive justice. Therefore, we argue, AI not only needs repair for the sake of environmental sustainability, but also for social and economic sustainability. Such repair first of all involves a different way of thinking about AI; instead of associating AI with automation, the common image of AI should be one that does not conceal the amount and variety of human labor involved. Secondly, repairing AI also implies transforming the global labor market linked to AI, in a way that better compensates and protects the AI workforce.

Another way to think of AI as requiring repair is with regards to the histories, intellectual traditions, and cultural perspectives that are neglected in AI development, AI governance, and debates about the ethics of AI. Some scholars have argued that AI upholds racial and colonial systems of oppression, and should therefore be *decolonized* (Adams, 2021; Cave & Dihal, 2020; McFadden & Alvarez, 2024; Mohamed et al., 2020). Such calls for decolonizing AI do not only apply to AI development, but also to AI ethics and AI governance. All of these practices, with their corresponding fields of research, are shaped predominantly by western perspectives and approaches. The issues that are given most attention in AI ethics debates and policy initiatives related to AI, reflect moral and social issues that are most applicable to members of western societies. Think about issues such as privacy, transparency, and trustworthiness. These are important issues, of course, but focus almost exclusively on the users of AI, which is only a select group of the global population – the ‘minority world’. Those who contribute to the creation of AI systems, such as gig workers or miners in the Global South, are overlooked. Moreover, because the AI companies that dominate the global market are mostly based in western countries (especially in the United States), working predominantly with developers and data from these parts of the world, AI applications only reflect the values, perspectives, and needs of a specific part of the world, even though the technology and its development affect the global population.

The histories, intellectual traditions, and cultural perspectives that have been neglected in the field of AI are not hidden in the same way as the materiality of AI and the human labor involved in AI are. However, they *are* hidden. These alternative views have been hidden through the reification of AI ideology. Reification entails that the origin of something is obscured and, instead, presented as a given or unavoidable matter (Lindgren, 2023). In the case of AI, reification means that it is generally not recognized that AI products and the AI industry reflect predominantly western, White, male, techno-solutionist, and profit-driven ideologies. Instead, *that* AI is developed and *how* it shapes our lives and societies, are taken for granted. Decolonizing AI, then, can be understood as a way of repairing AI. Decolonizing AI first of all changes how we think about AI, by revealing the ideology that has shaped AI. Secondly, decolonizing AI is aimed at actively transforming AI practices and debates, by making them more inclusive.

In summary, Stone and Van Wynsberghe (2024) have previously argued that making AI more sustainable requires repairing how we think about AI as well as how AI is developed. We argued in this section, that such repair is not only required on an environmental level, but also on a social and economic level. Repairing AI for social and economic sustainability entails, first of all, acknowledging the variety of human labor involved in developing AI (i.e., repairing how we, as a society, think about AI) and improving the socio-economic situation of AI workers (i.e., transforming AI infrastructures). Secondly, repairing AI entails decolonizing AI development, AI governance, and AI ethics, by revealing the ideologies behind these practices and debates (i.e., repairing how we think about AI) and by making them more inclusive (i.e., transforming AI development).

## AI Ideology

How AI is developed, and that it is developed in the first place, are not an inevitable given. Although it is often presented and perceived that way, technological development and technological progress are not predetermined necessities. *AI is ideological* – as we already pointed out briefly in the previous section. What this means is that AI is shaped by certain cultural, political, and economic agendas. It also implies that AI is *contingent* – following a different agenda, things could have been otherwise. The world took note with the discovery of embryonic stem cell research causing some countries to ban research on such tissues and others to move forward. What could have happened if some countries chose not to move forward with certain AI methodologies or applications?

Lindgren (2023) makes a distinction between the ‘ideology *behind* AI’ and ‘ideology *in* AI’, to point to the fact that ideology both shapes AI and is reinforced by AI. The AI products from companies such as OpenAI or Google, are strongly influenced by the neoliberal, predominantly male and White environment from which they originated. As a result, once AI has become an integral part of people’s lives and societies, the views and values representing the environment in which AI is created is reinforced by AI and all become subject to it. That AI reinforces certain ideologies not only leads to a homogenous culture that excludes different views and values, it has become painfully clear in recent years that the ideology behind and in AI also leads to discrimination (see for example Noble, 2018).

In line with Lindgren’s ‘ideology *in* AI’, Schütze (2024) argues that AI’s environmental impact goes beyond the energy, water, and mineral consumption involved in AI development. Schütze argues that AI ideology reproduces an unsustainable world. In other words, for Schütze, the sustainability of AI also has to do with the kind of world that this technology promotes. Brevini (2022) makes a similar argument, saying that AI encourages ‘uberconsumerism’, by constantly increasing the need for AI products, which in turn increases the need for datacenters, data, energy, water, minerals, and so on. Our addiction to and dependence on AI, social media, and other modern technologies, creates an endless spiral of consumption and resource depletion. Like other literature on the sustainability of AI, Schütze (2024) and Brevini (2022) are focusing solely on the fact that AI ideology reinforces an environmentally unsustainable world. In analogy with their arguments, we propose here that AI ideology contributes to socially and economically unsustainable structures as well.

Just like the AI industry places technological progress before the environment, it also prioritizes economic gains over social costs. The fast pace and massive scale of AI development, as well as the lavish profits made by the AI industry, are made possible through cheap, exploitative labor. While ‘tech bros’ in the Global North are rewarded generously for their labor, much of the human labor involved in AI is performed in developing countries or outsourced to those who find themselves struggling on the traditional labor market (e.g. single parents, people close to retirement age, disabled people, migrants, etc.). Moreover, as already pointed out in Sects. “The Two Branches of Sustainable AI” and “Repairing AI” above, these ‘AI workers’ not only perform cheap labor, most of the time they also lack legal protection, job security, health insurance, and the tasks they have to perform often pose serious risks to their physical and mental health.

Furthermore, proposed solutions to the problematic labor market behind AI are the same as the popular solution to the climate crisis: more technology. Just like many proposals to tackle climate change involve technological solutions, the *International Labor Organization* (no less!) reports that AI offers us an opportunity to mitigate the digital divide and promote inclusive and decent work for those working in the AI industry (ILO, 27 October 2023). AI is (too) often presented as a solution to problems that the AI industry itself created or contributed to. As Schütze (2024) rightfully points out, such techno-solutionism is yet another expression of AI ideology and therefore cannot entail a move away from that same ideology. Put differently, solving the problems caused by AI with more AI is like fighting fire with fire.

The ideology behind AI development, and embedded within the technology and its infrastructures, is a neoliberal one that favors free markets that serve the few, over socio-economic well-being for the many. It is the exploitation of human labor that makes it possible that tech companies like Amazon, Microsoft, Meta, and Alphabet, are among those with the highest revenues in the world (Murphy & Schifrin, 6 June 2024). This is not a new phenomenon, of course. It is a (neo)colonial pattern. It is imperative for the global AI community to re-think not only the environmental, but also the social and economic sustainability of current economic practices that shape and are shaped by the AI industry.

## A Structural Turn

So far, we have argued that sustainable AI debates and initiatives should not only be about the environmental sustainability of AI, but also include the social and economic sustainability of AI. However, we do acknowledge that current approaches and debates on the ethics of AI already, and increasingly so, consider the societal impact of AI. These considerations are for example reflected in debates about AI and fairness or AI and democracy. One may therefore ask: How is this case for considering the social and economic sustainability of AI adding to existing AI ethics debates? We address this question in this final section.

Similar to our intentions here, Rohde and colleagues (2024) recently argued for the inclusion of the social and economic pillars of sustainability in the notion of ‘sustainable AI’. However, where Rohde et al. (2024) focus on developing a framework for assessing the sustainability of AI, our focus in this article lies on further fleshing out arguments for the importance of considering the sustainability of AI. More importantly, the topics that Rohde and colleagues consider in relation to the social and economic sustainability of AI are different from the types of issues we have tried to highlight in this article. Under the header ‘social dimension’, Rohde et al. (2024, p. 4) place ‘transparency and accountability’, ‘nondiscrimination and fairness’, ‘technical reliability and human supervision’, ‘self-determination and data protection’, ‘inclusive and participatory design’, ‘cultural sensitivity’. Only the last item on this list addresses concerns that we have also raised, namely those addressed by the decolonial AI discourse. Under the header ‘economic dimension’, the authors consider ‘market diversity and exploitation of innovation potential’, ‘distribution effect in target markets’, and ‘working conditions and jobs’ (Rohde et al., 2024, p. 5). Again, only the last item on this list considers the types of issues we have discussed in this article – namely, the precarious and exploitative human labor involved throughout the AI lifecycle. While the items on their lists are important issues for AI ethicists to consider, we believe that the way in which Rohde et al. (2024) present the social and economic sustainability of AI is too close still to previous work done in AI ethics. Although we align on the position that the social and economic pillars of sustainability ought to be included in debates about sustainable AI, we think the authors do not demonstrate sufficiently how the consideration of the social and economic sustainability of AI can *add to* and *change* current debates and approaches in AI ethics and governance.

Previous debates on AI ethics were characterized by a focus on ethical principles and values that can be included in the design of AI products and guide AI developers. Notions that were central to AI ethics debates so far are ‘trustworthy AI’, ‘responsible AI’, ‘human-centered AI’, and ‘explainable AI’ (or ‘XAI’). Van Wynsberghe (2021) refers to these type of guidelines and design focused debates as “the second wave of AI ethics”. These debates concentrate predominantly the impact of AI products on users or on those living in the countries where AI is used most. We have shown in this article that hidden or excluded in AI ethics, so far, are the implications of AI and its infrastructures on those whose labor contributes to the realization of AI or those whose livelihoods, health, and living environments are affected by AI development (as also argued by Adams, 2024). In other words, while existing AI ethics principles and guidelines focus predominantly on AI’s impact on people in the Global North, considering the social and economic sustainability of AI, along with its environmental impact, would include the majority world as stakeholders in AI ethics debates. Hence, considering the social and economic sustainability of AI, along with its environmental costs, adds a new dimension to AI ethics and governance. 

Bolte and Van Wynsberghe (2024) argue that we can currently see a structural turn taking place in the AI ethics debate. They describe the second wave of AI ethics as “isolationist”, given its focus on the artefact-level, i.e., on specific AI applications. The new, third wave of AI ethics – which is well on its way – shifts the perspective to broader socio-economic questions regarding the infrastructures that constitute the AI domain. Bolte and Van Wynsberghe write that “the third wave of AI ethics rests on a turn towards a structural approach for uncovering ethical issues at a systemic level”, adding that “Sustainable AI is part of the third wave of AI ethics but does not constitute this third wave entirely” (2024, p. 8). In other words, the authors argue that the concern for sustainable AI is a symptom of a bigger trend, which is a focus on systems and structures in debates about AI ethics. Although they connect sustainable AI mainly to environmental concerns, like much of the literature on the sustainability of AI does, their argument would nevertheless apply to a broader notion of sustainable AI, like the one we presented in this paper.

Following Bolte and Van Wynsberghe’s 2024 analysis of the structural turn in AI ethics, we conclude that sustainable AI reflects a growing consensus within the AI ethics debate that all socio-economic costs connected to the global systems and infrastructures surrounding AI development need to be object of ethical analyses and policy making.

## Conclusion

The aim of this article was to argue that ‘sustainable AI’ should be about all three pillars of sustainability: the environment, economy, and society. While AI for sustainability initiatives have also considered AI’s use for socio-economic goals, discussions of the sustainability of AI are characterized thus far by a narrow focus on the environmental dimension of sustainability. This is a mistake, we argue, because considering the social and economic sustainability of AI development – such as the hidden labor involved in AI development, the impact of AI development on local economies and industries, and the ideological roots and impact of AI – adds a new and important perspective to ongoing debates about AI ethics and governance. Moreover, we have argued that the three pillars of sustainability are deeply intertwined, also in the AI context. Hence, to properly address the environmental costs of AI, the interrelated social and economic costs should be a part of the discussion.

This article is of an argumentative and conceptual nature. It is beyond the scope of the present discussion to formulate solutions to the problems raised. With our critique that the social and economic sustainability of AI have been overlooked, we do not wish to discredit the importance and urgency of addressing AI’s environmental costs. Instead, we simply wish to add issues to the agenda of AI developers, policy makers, and ethicists, and to shift the locus of our concerns from the users of AI systems, the minority world, to the global community that is affected by AI’s development processes and infrastructures. We conclude by stressing that action needs to be taken to change AI’s lifecycle and infrastructures in ways that make it not only more environmentally sustainable, but also imply a move towards more social and global justice.

## Data Availability

No data was used in developing this article.

## Footnotes

1 Although many of these types of labor are not exclusively performed for the development of AI only, they are nevertheless forms of ‘AI labor’, because they involve necessary steps to make the development and use of AI possible.
